# Seroepidemiology and Carriage of Diphtheria in Epidemic-Prone Area and Implications for Vaccination Policy, Vietnam

**DOI:** 10.3201/eid2901.220975

**Published:** 2023-01

**Authors:** Noriko Kitamura, Thanh T. Hoan, Hung M. Do, The A. Dao, Lien T. Le, Thao T.T. Le, Thuy T.T. Doan, Thuong N. Chau, Hoi T. Dinh, Masaaki Iwaki, Mitsutoshi Senoh, Androulla Efstraciou, Nen M. Ho, Duc M. Pham, Duc-Anh Dang, Michiko Toizumi, Paul Fine, Hung T. Do, Lay-Myint Yoshida

**Affiliations:** London School of Hygiene and Tropical Medicine, London, UK (N. Kitamura, P. Fine);; Nagasaki University, Nagasaki, Japan (N. Kitamura, M. Toizumi, L.-M. Yoshida);; Pasteur Institute in Nha Trang, Nha Trang, Vietnam (T.T. Hoan, H.M. Do, T.A. Dao, L.T. Le, T.T.T. Le, T.T.T. Doan, H.T. Do);; Quang Ngai Provincial Health Services, Quang Ngai, Vietnam (T.N. Chau, H.T. Dinh, N.M. Ho, D.M. Pham);; National Institute of Infectious Diseases, Tokyo, Japan (M. Iwaki, M. Senoh);; National Institute of Hygiene and Epidemiology, Hanoi, Vietnam (D.-A. Dang);; UK Health Security Agency, London (A. Efstraciou)

**Keywords:** diphtheria, seroepidemiology, carriage, Corynebacterium species, bacteria, epidemic-prone area, vaccine, vaccination policy, immunity, disease outbreaks, Vietnam

## Abstract

In 2019, a community-based, cross-sectional carriage survey and a seroprevalence survey of 1,216 persons 1–55 years of age were conducted in rural Vietnam to investigate the mechanism of diphtheria outbreaks. Seroprevalence was further compared with that of an urban area that had no cases reported for the past decade. Carriage prevalence was 1.4%. The highest prevalence, 4.5%, was observed for children 1–5 years of age. Twenty-seven asymptomatic *Coerynebacterium diphtheriae* carriers were identified; 9 carriers had *tox* gene–bearing strains, and 3 had nontoxigenic *tox* gene–bearing strains. Child malnutrition was associated with low levels of diphtheria toxoid IgG, which might have subsequently increased child carriage prevalence. Different immunity patterns in the 2 populations suggested that the low immunity among children caused by low vaccination coverage increased transmission, resulting in symptomatic infections at school-going age, when vaccine-induced immunity waned most. A school-entry booster dose and improved infant vaccination coverage are recommended to control transmissions.

Diphtheria is an infectious disease caused by toxigenic strains of *Corynebacterium diphtheriae, C. ulcerans*, and, rarely, *C. pseudotuberculosis* (*1*–*3*). Although the diphtheria toxoid vaccine contributed to a decrease in the number of diphtheria cases globally, the disease remains a threat to public health, particularly in South and Southeast Asia (*4*,*5*).

Currently, the World Health Organization recommends 3 primary doses of the diphtheria‒tetanus‒pertussis (DTP) vaccine in young infants (i.e., at 6, 10, and 14 weeks of age), followed by 3 booster doses at 12–23 months, 4–7 years, and 9–15 years of age, to protect all age groups. Nevertheless, many low- and middle-income countries have not introduced all booster doses.

The Vietnamese Ministry of Health (MOH) first introduced DTP in 1981, targeting infants 2, 3, and 4 months of age. A booster dose targeting children 18 months of age was introduced during 2011 (*6*). Because of efforts in vaccination, reported diphtheria cases in Vietnam decreased to nearly zero by 2010. However, several small diphtheria outbreaks in remote districts in central and western Vietnam have been observed since 2013 (*7*).

Supplemental immunization activities (SIAs), in which vaccination is delivered to all targeted persons regardless of their previous vaccination history, were conducted in the areas surrounding Quang Ngai Province when diphtheria cases were identified during 2013‒2019 (*8*). However, most of the population of Quang Ngai Province has not been covered by SIAs as of October 2019. According to the national surveillance program, Quang Ngai Province reported 2 laboratory-confirmed cases in 2017–2018 and 47 in 2019–2020, among an estimated population of 1,231,697 (*9*). Among these cases, 36 (73%) cases were in school-age children (6–17 years of age). Among confirmed cases, 3 (6%) were fatal.

Although national administrative coverage of 3 doses of DTP among infants has been maintained above 90% in Vietnam since 1994 (excluding 2002 and 2013), subnational coverage has not always been high (*10*). In addition, although low vaccination coverage in localized spots appeared to cause diphtheria outbreaks, the immune profile of the population in these areas is unknown (*4*). The World Health Organization suggests including adults in SIAs to control diphtheria outbreaks because adults might also be susceptible. However, no specific age groups are recommended because epidemiologic characteristics differ by country (*2*).

Asymptomatic carriers play a major role in transmission dynamics, but details of the carrier stage in affected areas are largely unknown because the proportion of healthy carriers who carry toxigenic and nontoxigenic strains has not been investigated in Southeast Asia (*11*,*12*). Moreover, host factors that govern carriage status have not been elucidated.

This study aimed primarily to measure the carriage prevalence of *Corynebacterium* species in the respiratory tract in areas in which outbreaks occurred and to assess potential risk factors for carriage. The second aim was to measure the age-stratified serologic immune profile against diphtheria toxin to help to identify the mechanism of the recent outbreaks and target the most appropriate age groups for SIA. Reflecting a previous study suggesting that low antibody levels increased the risk for being a carrier (*13*), this study also examined the factors that contributed to low immunity among persons. The third aim was to compare the immune profile patterns in areas in which cases have been reported and not reported to discuss the current DTP schedule in Vietnam.

## Methods

### Study Site

**Figure 1:**
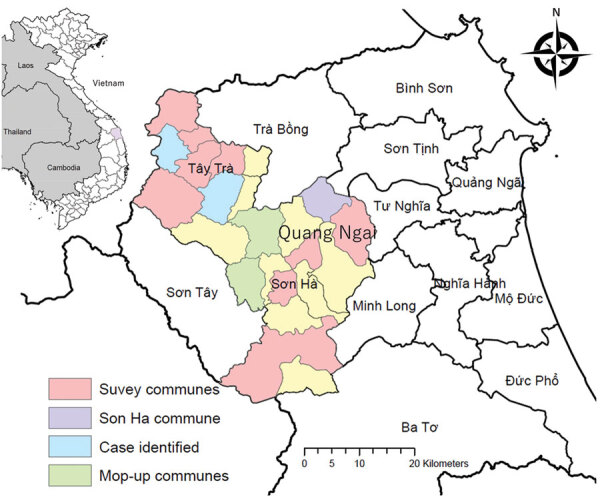
Study areas and locations where the cases were identified before and during diphtheria study in Tay Tra and Son Ha districts in Quang Ngai Province, Vietnam. Red and purple indicate 10 communes selected for this study. Blue and purple indicate 1 laboratory-confirmed diphtheria case reported during January‒September 2019 in each of these communes. Purple (Son Ha commune) indicates 12 confirmed cases reported in this commune within 1 month from the survey date, October 2019. Green indicates 2 communes excluded from the selection process of this study because a mop-up vaccine campaign was conducted in 2018. Inset map shows location of the study area in Vietnam.

Two districts, Tay Tra and Son Ha in Quang Ngai Province, were selected as a study area because 3 diphtheria cases were identified there during January‒September 2019, and no SIAs had been implemented (Figure 1). Two communes in the Son Ha District were excluded because a mop-up vaccination campaign of DTP was conducted in those communities during 2018. The estimated population of the 2 study districts was 99,121 in 2019 (*9*). Health access is limited in this area because of the mountainous topography.

### Study Design and Sampling Method

We conducted a community-based cross-sectional survey in October 2019. We stratified age into 4 groups, 1–5, 6–17, 18–40, and 41–55 years of age; children attend primary through high school between the ages of 6 and 17 years in Vietnam. On the basis of previously obtained age-stratified seroprevalence in Vietnam (*14*,*15*), the required sample size for each age stratum was estimated to be 350, 400, 400, and 350, respectively, with 10% precision, a 3.5 design effect, and an 80% response rate.

We conducted multistage cluster sampling. In each district, we sampled 5 communes by population proportion to size and selected 3 villages from each commune by simple random sampling. A total of 30 villages were selected (Figure 1). Because the average household size in Vietnam is 4 persons (*16*), we selected 15 households in each village and 450 households to recruit 1,500 persons. We oversampled households that had children 1–5 years of age to recruit a higher proportion of the sample size than the original population.

### Data and Sample Collection and Ethics

Local healthcare workers visited homes of participants to invite them to participate in the survey. Written informed consent was obtained from each participant or guardian. The survey teams interviewed each participant by using a standardized questionnaire and collected sociodemographic information. Based on previously reported risk factors for diphtheria infection or carriage of *C. diphtheriae*, age, vaccination history, seropositivity (diphtheria toxoid IgG titer >0.1 IU/mL), bed-sharing, school attendance, staying in school dormitories, household size, frequency of bathing or handwashing, having livestock or companion animals, diphtheria toxoid IgG level, and mid-upper arm circumference (MUAC) were assessed for their association with carriage of *Corynebacterium* species (*13*,*17*–*21*). MUAC was used as a measure of the nutritional status of children 1–5 years of age. We collected vaccination history for children <10 years of age from either the vaccination card of the participant or the vaccine registration book at the respective community health centers in their residence area.

We collected dried blood spots (DBS) by using venipuncture or fingerprick onto 903 protein saver cards (#Z761575; Whatman, https://www.cytivalifesciences.com/en/us/about-us/our-brands/whatman) and stored them at −80°C according to the procedure of the Centers for Disease Control and Prevention (*22*,*23*). We collected throat swab specimens and stored them in Amies medium and collected nasopharyngeal swab specimens and stored them in skim milk, tryptone, glucose, and glycerin medium (*3*). All collected samples were stored at the Pasteur Institute in Nha Trang and stored at −80°C until testing. Ethics approval was obtained from the ethical review boards of the Pasteur Institute in Nha Trang, MOH Vietnam, Nagasaki University, and the London School of Hygiene and Tropical Medicine (1775/IPN-DT, 1046/K2DT-KHCN, Nagasaki University Institutional Review Board approval no. 191226228, LSHTM ethics reference no. 17518).

### Microbiological Tests

We cultured collected swab specimens on tellurite-containing agar medium in a 35°C incubator for 24‒48 hours (*3*). If black colonies grew, we initially tested them by Gram stain to identify gram-positive bacilli (*3*). We used the API Coryne Test (bioMérieux, https://www.biomerieux.com) to identify species and biovars for each subculture (*3*). We tested subcultures for expression of the diphtheria toxin by using the modified Elek test (*24*).

We conducted quadruplex, real-time, reverse transcription PCR (qRT-PCR) directly on throat swab specimens and aliquots of skim milk, tryptone, glucose, and glycerin medium to identify *C.diphtheriae*, *C.ulcerans*, or *C. pseudotuberculosis* and the diphtheria toxin gene according to published methods (*3*,*25*). DNA was extracted by using the QIAmp DNA Extraction Kit (QIAGEN, https://www.qiagen.com) (*26*). Primers and probes targeted 2 *rpoB* genes, the *tox* gene, and the green fluorescent protein gene (*gfp*), which we used as internal positive controls.

### Diphtheria Toxoid Serologic Assay

We measured diphtheria toxoid IgG levels by using a commercially available ELISA Kit (Binding Site, https://www.bindingsite.com) according to the manufacturer’s protocol. We punched out a DBS with a 6-mm hole punch and stored in Eppendorf tubes, then eluted DBS with 500 μL elution buffer and incubated overnight at 4°C. We then used the supernatant of the eluted solution for the ELISA (*27*–*30*). We defined an IgG level >0.1 IU/mL, an international standard cutoff value, as seropositive (*31*,*32*).

### Comparison of Seroprevalence in 2 Areas with or without Reported Cases

This study compared seroprevalence in an epidemic-prone area (Quang Ngai Province) and a nonepidemic area in Vietnam. Regarding the nonepidemic area, Nha Trang (city) in Khanh Hoa Province was selected because the population is well-vaccinated and has not reported any diphtheria cases since 2013. Moreover, the age-stratified seroprevalence data among persons 1–55 years of age were investigated in Nha Trang during 2017 (*15*). Therefore, we compared the immunity pattern of the population in Quang Ngai Province with that for Nha Trang.

**Figure 2:**
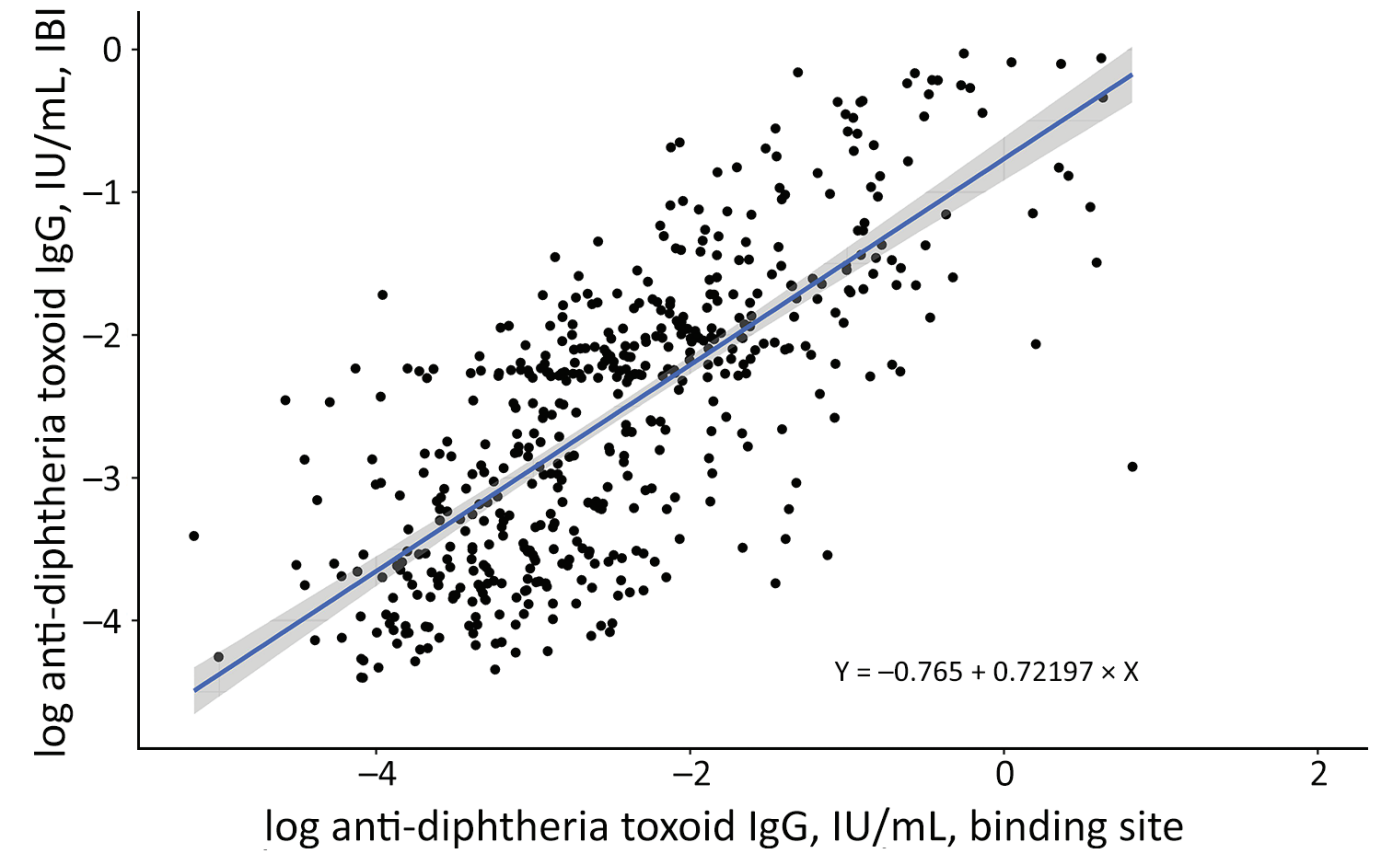
Best-fitted linear regression line (blue line) comparing log-transformed IgG concentrations measured by Binding Site and IBL ELISAs. Shaded region indicates 95% CI.

We used 2 different ELISAs for measuring diphtheria toxoid IgG: the Binding Site ELISA for the study in Quang Ngai and the IBL ELISA (https://www.ibl-international.com) for the study in Nha Trang. First, we tested 546 subsets of the samples collected in Quang Ngai by using 2 ELISA kits in parallel and compared the 2 results by using linear regression analysis. On the basis of the best-fitted line, we converted the log-transformed IgG value measured by the Binding Site test to the value of IBL by using the equation Y(log IgG IBL) = −0.7652 + 0.72197X (log IgG Binding Site) (Figure 2).

We recalculated seroprevalence for Quang Ngai by using the converted IgG concentration and stratified seroprevalence into 5 age groups of 1–5, 6–15, 16–25, 26–35, and 36–55 years. We compared age-stratified seroprevalence and 95% CIs in Quang Ngai and Nha Trang.

### Statistical Analysis

We measured carriage prevalence and seroprevalence by using 95%s CIs after being weighted by population size. We summarized sociodemographic information on the participants by districts. We examined differences in characteristics of the districts by using the χ^2^ test or t-test.

We used the Fisher exact test or *t-*test to examine the association between carriage status and each risk factor. We conducted multivariate logistic regression to confirm whether carriage status was associated with young persons or persons who had low levels of IgG. Because nutrition is a critical element for immunoresponse, we conducted multivariate linear regression to explore the association between immunity level (natural log-transformed IgG) and nutrition status (MUAC) of a person with adjustment for age. We conducted statistical analyses by using Stata 15 (*33*).

## Results

We recruited 1,216 persons from 458 households. Of those, 269 (22%) were 1–5 years of age, 322 (26%) 6–17 years of age, 523 (43%) 18–40 years of age, and 102 (8%) 41–55 years of age; 615 (51%) were male and 601 (49%) female. Of children <10 years of age, 75% had received >1 dose, 74% had received 3 doses, and 43% had received 4 doses of DTP (DTP1, DTP3, and DTP4). There was no statistical difference in DTP3 or DTP4 coverage between the 2 districts. No participants recalled any symptom or diagnosis of diphtheria in the past. Nobody had received DTP or tetanus‒diphtheria vaccine because of injuries or involvement in the recent SIAs. Eighty percent of participants in Tay Tra District were in the Co ethnic group, and 87% of participants in Son Ha District were in the Hre ethnic group. The major ethnic group in Vietnam, the Kinh, accounted for only small proportions in the 2 districts. Most (91%) of the adult participants were farmers (Table 1).

**Table 1:** Sociodemographic characteristics of participants and households in Tay Tra and Son Ha District in survey, Quang Ngai Province, Vietnam, 2019*

Individual data	All, n = 1,216	Tay Tra, n = 604	Son Ha, n = 612	p value
Age, y				
<5	269 (22)	125 (21)	144 (24)	0.45†
6‒17	322 (26)	171 (28)	151 (25)	
18‒40	523 (43)	258 (43)	265 (43)	
40‒55	102 (8)	50 (8)	52 (8)	
Sex				
M	615 (51)	309 (51)	306 (50)	0.69†
F	601 (49)	295 (49)	306 (50)	
Ethnic group				
Co	487 (40)	486 (80)	1 (0.2)	<0.01†
Hre	531 (44)	0 (0)	531 (87)	
K’Dong	110 (9)	105 (17)	5 (1)	
Kinh	79 (6)	5 (1)	74 (12)	
Other	9 (1)	8 (1)	1 (0.2)	
>18 years old	All, n = 625	Tay Tra, n = 308	Son Ha, n = 317	
Occupation				
Farmer	569 (91)	278 (90)	291 (92)	0.50†
Other	56 (9)	30 (10)	26 (8)	
<10 years old	All, n = 464	Tay Tra, n = 231	Son Ha, n = 233	
Confirmed vaccination history				
BCG	361 (78)	165 (71)	196 (84)	<0.01†
DTP1	347 (75)	165 (71)	182 (78)	0.12†
DTP3	343 (74)	163 (71)	180 (77)	0.12†
DTP4	198 (43)	90 (39)	108 (46)	0.13†
Measles	350 (75)	160 (69)	190 (82)	0.02†
MUAC, cm	All, n = 235	Tay Tra, n = 111	Son Ha, n = 124	
Mean (SD)	14.7 (1.3)	14.6 (1.4)	14.8 (1.2)	0.20‡
Household data	All, n = 458	Tay Tra, n = 252	Son Ha, n = 206	
Toilet facility	323 (71)	215 (85)	108 (52)	<0.01§
Water source				
Well	168 (37)	2 (1)	166 (81)	<0.01§
River	249 (54)	247 (98)	32 (16)	<0.01§
Energy source				
Gas	95 (21)	22 (9)	73 (35)	<0.01§
Bio fuel	358 (78)	226 (90)	132 (64)	<0.01§

*Values are no. (%) except as indicated. BCG, *Mycobacterium bovis* Bacillus Calmette–Guérin; DTP, diphtheria‒tetanus‒pertussis; MUAC, mid-upper arm circumference. †By χ^2^ test. ‡By t-test. §By χ^2^ test or Fisher exact test.

Overall weighted carriage prevalence of *Corynebacterium* species was 1.4% (95% CI 0.4%–5.3%), and the prevalence of the *tox* gene–bearing strain was 0.5% (95% CI 0.0%–4.7%). Age-stratified carriage prevalence levels were 4.5% for those 1–5 years of age, 2.5% for 6–17 years of age, 1.0% for 18–40 years of age, and 0.0% for 41–55 years of age. Overall weighted seroprevalence of diphtheria toxoid IgG (>0.1 IU/mL) in the study area was 51% (95% CI 44%–59%). Age-stratified seroprevalence levels were 40% for those 1–5 years of age, 37% for 6–17 years of age, 55% for 18–40 years of age, and 63% for 41–55 years of age (Table 2).

**Table 2:** Age-stratified carriage prevalence of *Corynebacterium diphtheriae* and seroprevalence of diphtheria toxoid IgG () in 2 districts, Quang Ngai Province, Vietnam*

Group	Mean ± SD age, y	Total.	Seroprevalence, No. (%) 95% CI		Carriage prevalence, No. (%) 95% CI
Age group, y					
<5	3.2 ± 1.36	269	108 (39.5) 22.7‒59.2		12 (4.50) 3.7‒5.5
6‒17	10.1 ± 3.17	332	120 (36.7) 29.4‒44.6		10 (2.5) 0.1‒47.5
18‒40	29.5 ± 5.61	513	283 (55.3) 42.8‒67.1		5 (1.0) 0.6‒1.7
41‒55	46.3 ± 4.30	102	64 (62.9) 60.9‒64.8		0 (0) NA
Total	20.0 ± 14.3	1,216	575 (51.4) 3.6‒59.1		27 (1.4) 0.4‒5.4
District					
Tay Tra, by age group, y					
<5	3.1 ± 1.42	125	41 (32.8) 25.1‒41.5		6 (4.8) 2.2‒10.3
6‒17	9.9 ± 3.09	171	59 (34.5) 27.8‒41.9		0 (0) NA
18‒40	29.7 ± 5.19	258	152 (58.9) 52.8‒64.8		3 (1.2) 0.4‒3.5)
41‒55	46.4 ± 4.69	50	31 (62.0) 47.9‒74.3		0 (0) NA
Total	20.0 ± 14.2	604	283 (50.6) 33.6‒67.5		9 (0.9) 0.2‒3.7
Son Ha, by age group, y					
<5	3.2 ± 1.31	144	67 (46.5) 38.5‒54.7		6 (4.2) 1.9‒9.0
6‒17	10.4 ± 3.25	151	61 (40.4) 32.9‒48.4		10 (6.6) 3.6‒11.9
18‒40	29.4 ± 6.04	265	131 (49.4) 43.4‒55.4		2 (0.8) 0.2‒0.3
41‒55	46.2 ± 3.94	52	33 (63.5) 49.7‒75.3		0 (0) NA
Total	20.0 ± 14.4	612	292 (52.4) 39.1‒65.3)		18 (2.0) 0.3‒11.0

*Seroprevalence and carriage prevalence were weighted for overall groups. IgG cutoff >0.1 IU/mL. NA, not applicable.

We identified 27 carriers of *C. diphtheriae* by qRT-PCR. Among identified carriers, 17 (63%) were female (10 1–5 years of age) and 10 (37%) male (2 1–5 years of age). Sixteen carriers had received >3 doses of DTP. *C. diphtheriae* was isolated by culture from 17 of 27 qRT-PCR–positive samples. Eleven samples were biovar *mitis,* and 6 were biovar *gravis*. Swab specimens from 9 of the 27 carriers (33%) were *tox* gene positive by qRT-PCR, but only 6 specimens were successfully recovered by isolation. From those 6 isolates, diphtheria toxin expression was confirmed in 3 isolates by using the modified Elek test (2 biovar *mitis* and 1 biovar *gravis*). The remaining 3 isolates did not express diphtheria toxin and were thus *tox* gene–bearing nontoxigenic strains (NTTB). All 3 belonged to biovar *mitis* (Table 3).

**Table 3:** Geographical distribution, characteristics, and vaccination history of 27 carriers of *Corynebacterium diphtheriae,* Vietnam*

Patient no.	District	Commune	Village	HH ID no.	Age, y/sex	*tox* gene	Biovar	Elek test result	Vaccine status, no. doses	DTP3 coverage, % (95% CI)
1	Tay Tra	Tra Phong	Tra Nga	305	25/M	‒	*gravis*	‒	NA	60 (35‒81)
2		Tra Thanh	Thon Mon	818	23/M	‒	NA	NA	NA	76 (1‒91)
3†		Tra Lanh	Tra Luong	1003	40/F	‒	*mitis*	‒	NA	64 (34‒86)
4		Tra Xinh	Tra Kem	1401	5/F	‒	NA	NA	4	85 (55‒96)
5				1404	2/F	‒	NA	NA	4	
6				1407	4/F	‒	NA	NA	4	
7			Tra Veo	1501	3/F	‒	mitis	‒	4	63 (38‒82)
8					5/F	‒	mitis	‒	4	
9				1505	2/F	+	mitis	‒	4	
10	Son Ha	Son Ha	Deo Ron	1607	10/F	+	NA	NA	0	71 (44‒89)
11					3/F	‒	*mitis*	‒	3	
12					2/F	+	NA	NA	4	
13			Dong Reng	1704	9/M	‒	NA	NA	0	40 (19‒65)
14				1707	14/M	+	*mitis*	+	NA	
15			Ha Bac	1807	9/M	+	NA	NA	0	71 (46‒87)
16				1811	7/F	+	*mitis*	+	4	
17					10/M	+	*mitis*	‒	3	
18				1812	10/M	+	*mitis*	‒	3	
19					4/M	‒	*mitis*	‒	0	
20		Son Giang	Lang Ri	2314	10/F	‒	*mitis*	‒	0	94 (68‒99)
21			Ta Dinh	2406	28/M	‒	*gravis*	‒	NA	93 (63‒99)
22					25/F	+	*gravis*	+	NA	
23				2409	3/M	‒	*gravis*	‒	4	
24		Son Ky	Lang Re	2505	7/F	‒	NA	NA	4	88 (61‒97)
25		Son Hai	Lang Trang	3004	7/F	‒	*gravis*	‒	3	80 (57‒92)
26				3011	2/F	‒	NA	NA	3	
27				3012	5/F	‒	*gravis*	‒	3	

*DTP3 coverage was the coverage at the village level where carriers were living. DTP, diphtheria‒tetanus‒pertussis; HH, household; ID, identification; NA, not available; ‒, negative; +, positive. †For patient 3, *C.diphtheriae* was identified from a nasopharyngeal swab specimen and a throat swab specimen. Others were identified only from nasopharyngeal swab specimens.

We identified 27 carriers from 21 households located in 8 communes. Ten of 27 lived in a commune known as the Son Ha commune. Of 21 households, >1 carriers were identified in 5 households. Four households had 2 carriers, and 1 household had 3 carriers (Table 3).

We found strong evidence that age and IgG level were associated with carriage status (Table 4). Young children were likely to be carriers, after adjusting for IgG levels. High IgG levels were unexpectedly associated with carriers after adjusting for age. Multivariate linear regression analysis showed that smaller MUAC was associated with low IgG levels after adjusting for age, although MUAC was not associated with carriage status (Tables 4, 5).

**Table 4:** Associations between *Corynebacterium diphtheriae* carriage and potential risk factors, Vietnam*

Risk factor	Carriage, no. (%)		p value†
	Total	Tested	
Age group, y			
<5	12 (4.5)	257 (95.5)	<0.01
6‒17	10 (3.1)	314 (96.9)	
18‒40	5 (1.0)	518 (99.0)	
41‒55	0	102 (100.0)	
Sex			
M	10 (1.6)	605 (98.4)	0.198
F	17 (2.8)	584 (97.2)	
DTP1 <10 years			
0 doses	5 (4.5)	107 (95.5)	>0.99
>1 dose	16 (4.5)	336 (95.5)	
DTP3 <10 years			
<3 doses	5 (4.3)	111 (95.7)	>0.99
>3 doses	16 (4.6)	332 (95.4)	
Diphtheria antibody, IU/mL			
<0.1	11 (1.7)	630 (98.3)	0.24
>0.1	16 (2.8)	559 (97.2)	
School			
Not attended	17 (1.8)	906 (98.2)	0.12
Attended	6 (2.1)	283 (97.9)	
Dormitory			
Not staying	23 (2.2)	1,035 (97.8)	0.77
Staying	4 (2.5)	154 (97.5)	
Sharing bed			
Yes	4 (2.7)	143 (97.3)	0.56
No	23 (2.2)	1,037 (97.8)	
Household size, no, persons			
<4	13 (2.2)	585 (97.8)	>0.99
>4	14 (2.3)	604 (97.7)	
Bathing, times/day			
<1	0	72 (100.0)	0.40
>1	27 (2.4)	1,117 (97.6)	
Handwashing, times/day			
<3	4 (1.6)	247 (98.4)	0.11
>3	18 (3.9)	445 (96.1)	
Livestock or pet animal			
No	24 (2.7)	866 (97.3)	0.08
Yes	3 (0.9)	323 (99.1)	
Category	Positive	Negative	p value by t-test
MUAC, cm, mean (SD)	15.0 (1.77)	14.8 (1.33)	0.16
Age, y, mean (SD)	7 (9.7)	20 (14.3)	<0.01
log-transformed IgG level, mean (SD)	‒1.4 (2.1)	‒2.2 (1.2)	<0.01

*DTP, diphtheria‒tetanus‒pertussis; MUAC, mid-upper arm circumference. †By Fisher exact test except as indicated.

**Table 5:** Associations between *Corynebacterium diphtheriae* carriage and diphtheria toxoid IgG levels adjusted for age assessed by logistic regression and between IgG levels and mid-upper arm circumference adjusted for age by linear regression, Vietnam*

Association between carriage status and IgG	Crude odds ratio 95% CI)	p value	Adjusted odds ratio (95% CI)	p value
log-transformed IgG level	1.49 (1.15‒1.93)	<0.01	1.49 (1.17‒1.90)	<0.01
Age, y	0.94 (0.90‒0.97)	<0.01	0.94 (0.90‒0.97)	<0.01
Association between IgG and MUAC	Crude coefficient	p value	Adjusted coefficient	p value
MUAC, cm	0.01 (‒0.01 to 0.02)	0.43	0.02 (0.00‒0.03)	0.014
Age, y	‒0.21 (‒0.34 to ‒0.08)	<0.01	‒0.31 (‒0.45 to ‒0.16)	<0.01
*MUAC, mid-upper arm circumference.				

**Figure 3:**
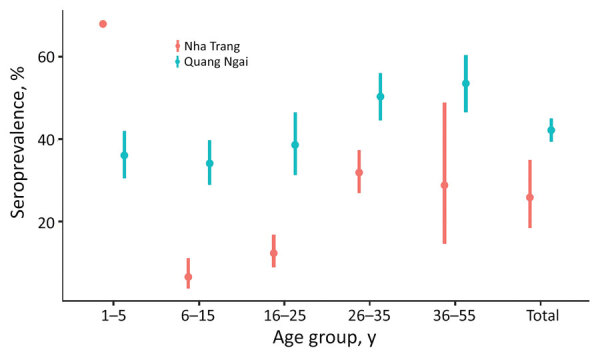
Comparison of the age-stratified seroprevalence, the proportion of persons who had diphtheria toxoid antibody >0.1 IU/mL, between Quang Ngai Province and Nha Trang City (15), Vietnam. Seroprevalence of Quang Ngai was not weighted by population for this comparison. Nha Trang is a well-vaccinated community that has had no reported diphtheria cases since 2013. Error bars indicate 95% CIs.

The overall seroprevalence was higher for Quang Ngai (47%, 95% CI 45%–50%) than for Nha Trang (26%, 95% CI 20%–32%). The seroprevalence among children 1–5 years of age was lower in Quang Ngai (36%, 95% CI 31%–42%) than in Nha Trang (68%, 95% CI 67%–69%), and the seroprevalence among children 6–15 years of age in Quang Ngai (34%, 95% CI 29%–40%) was higher than in Nha Trang (7%, 95% CI 4%–11%) (Table 6; Figure 3).

**Table 6:** Associations between *Corynebacterium diphtheriae* carriage and age group in 2 districts, Vietnam

Age, y	Na Trang, % (95% CI)	Qung Ngi, % (95% CI)
1–5	68 (67–69)	36 (31–42)
6–10	7 (4–9)	34 (29–40)
16–25	12 (7–19)	39 (31–46)
25–36	33 (27–40)	50 (47–56)
36–55	28 (17–43)	54 (47–60)
Total	26 (20–32)	42 (39–46)

## Discussion

We conducted this study to investigate the potential mechanisms underlying the recent outbreaks of diphtheria in Vietnam and to recommend a reasonable outbreak response and vaccination strategy. This study described community-based *C. diphtheriae* carriage prevalence in a diphtheria epidemic-prone area and assessed potential risk factors for carrier status and low immunity among persons. We also highlighted the difference in population immunity between the diphtheria epidemic-prone and nonepidemic areas.

The carriage prevalence, especially the prevalence of toxigenic strains, in the study population was much higher than that reported recently in Europe. According to a multicountry study conducted in Europe during 2007–2008, the prevalence of toxigenic strain in 8 countries in Europe was 0% (*34*). Toxigenic strains were isolated only in Latvia (0.08%) and Lithuania (0.07%), which had >1,500 cases and 112 cases reported, respectively, since 1994 (*35*,*36*). The prevalence of nontoxigenic strain was reported as 0.4% in Turkey in the same study (*34*). In our study, carriage prevalence was highest in the youngest age group and decreased by age. In Italy, 0.15% of healthy children 6–14 years of age carried a nontoxigenic strain in the early 2000s (*37*). In Indonesia, the prevalence of a toxigenic strain was reported as 3% among children 1–15 years of age during the outbreak in 2012 (*38*). Based on these findings, the long-running child vaccination program in Europe appears to have reduced carriage prevalence, especially the carriage prevalence of toxigenic strains. Conversely, toxigenic strains were still identified in countries in which symptomatic cases have been reported in the past 10 years. In addition, the current carriage prevalence in this study was similar to the situation in the United Kingdom during 1971 (1.2%) (*39*). If one considers that introduction of DTP in the United Kingdom was during 1941, forty years earlier than in Vietnam, vaccination coverage might be required to be adequate in the next few decades to reduce the carriage prevalence of toxigenic strain in Vietnam. The high prevalence of toxigenic strain indicates that more cases might be observed if the population remains susceptible. Twelve additional laboratory-confirmed cases were identified within 1 month from the survey date in Son Ha commune in which the highest carriage prevalence was observed.

Nine (33%) of 27 carriers harbored *tox* gene‒bearing strains. The remaining 18 were nontoxigenic strains, which rarely cause invasive diseases (*40*,*41*). Conversely, nontoxigenic strains were often suggested to play a major role in maintaining the transmission of *C. diphtheriae* among human hosts (*12*,*42*). Nontoxigenic strains could be converted to toxigenic ones by lysogenization with a specific temperate bacteriophage. Lysogenic conversions might occur in nontoxigenic strains in carriers, and the converted strains might infect others (*43*). Multilocus sequence typing of the identified strains from carriers and cases in this study might provide evidence to indicate that this conversion might have occurred in this community.

For 9 *tox* gene‒bearing strains, all 3 healthy carriers who had a nontoxigenic *tox* gene‒bearing (NTTB) strain have received 3 doses of DTP, which supports that NTTB strains are increasingly identified in Europe because of vaccine pressure (*11*,*44*). The current vaccine does not protect persons from NTTB strains (*45*). Although it is unlikely that NTTB strains will be an immediate threat in Vietnam, it might be necessary to monitor NTTB strains as a potential cause of disease in the future.

We found that carriers were concentrated in specific households and communities. This observation was consistent with household transmission being the main route of *C. diphtheriae* transmission in the prevaccination era (*46*). Once diphtheria appears in a household or specific community, transmissions might continue if persons in neighboring areas are not well vaccinated (*43*).

We found no association between carrier status and bed-sharing, staying at the school dormitory, or less frequent bathing, but several other studies have identified those as risk factors for infection (*17*–*21*). A small number of carriers might be a reason that we could not identify the association; biologic characteristics, such as age or individual immunity level, might have been played a greater role than social factors. At an aggregated level, carriage prevalence was negatively associated with seroprevalence against diphtheria. However, we could not identify the association between carrier status and low IgG level at an individual level, probably because of natural boosting of immunity after being a carrier. Because this study was cross-sectional, we could not prove the chronological change in immunity and carriage status for an individual directly.

We confirmed the lowest seroprevalence was in persons 6–17 years old (37%) because it was expected from the previous findings that most of the laboratory-confirmed cases were of school-going age (*7*). In addition, the seroprevalence was similarly low among children 1–5 years of age (40%), which might occur because of low DTP3 coverage and the waning of vaccine-derived immunity. Another potential reason is that the seroconversion rate after DTP vaccination might have been low because of host factors, such as malnutrition or external factors, such as suboptimum cold chains. In Quang Ngai Province, it was reported that 5.7% of children <5 years of age were wasted, and 25.5% were stunted in 2013 (*47*). Because small MUAC was associated with low levels of diphtheria toxoid IgG, poor nutrition status might be associated with low immunoresponse in persons.

The age-stratified seroprevalence for Quang Ngai Province compared with that for Nha Trang provided insights of waning and acquired immunity. The seroprevalence among persons 1–5 years of age in Quang Ngai was lower than that in Nha Trang, most likely because of the low vaccination coverage in Quang Ngai. Conversely, the seroprevalence among those 6–15 years of age in Quang Ngai was higher than that in Nha Trang, potentially reflecting the continuous natural exposure in Quang Ngai. This observation indicates that the low immunity among children 1–5 years of age led to ongoing transmission, resulting in high seroprevalence among those >6–15 years of age in Quang Ngai than in Nha Trang. The same observation was found in a seroprevalence survey in Indonesia during 2012 (*38*).

This study compared the IgG levels measured in DBS and serum. Schou et al. reported a good correlation for diphtheria serum DBS (*28*). In addition, we compared the diphtheria antibody levels measured by using the same ELISA kit (IBL) for serum samples and DBS by using 96 samples collected in Vietnam. We found high sensitivity (0.91) and specificity (0.92) of seropositive of age, when vaccine-induced immunity showed the greatest decrease. Persons >17 years of age were more protected than young age groups, probably by naturally acquired immunity. Nevertheless, 50% of the population >17 years of age were susceptible, which explains why all age groups have been recently affected by diphtheria (*4*). A school-entry booster dose will be recommended to prevent future cases because the infant immunization program appeared to create low immunity in school age children (*15*). Conversely, low immunity in preschool age children would be another reason for the recent epidemic in Quang Ngai Province. Therefore, improving routine infant vaccination coverage will be essential to control diphtheria.

Based on the low seroprevalence in the age groups 1–5 and 6–17 years of age, SIAs would be most effective if they targeted the population 1–17 years of age. The Vietnamese MOH so far included the population 1–40 years of age a target of diphtheria SIAs, but SIAs in Indonesia, Bangladesh, and Haiti targeted children 1–14 years of age (*48**–**50*). In Vietnam, targeting the population of 18–40 years of age could be beneficial because 50% of this age group was susceptible. Also, we should also be aware that SIAs would not stop transmission in a short time once transmission has started in susceptible populations.

This study found that 1.4% of the population were healthy carriers of *C. diphtheriae*. Two-thirds of them harbored nontoxigenic strains, which could be transmitted among human hosts asymptomatically. A school-entry booster dose and improved infant vaccination coverage are recommended to stop current *C.diphtheriae* transmission in Vietnam. SIAs targeting persons 1–17 years of age will be efficient as an outbreak response.
